# PP-2024-44 Why Early Savings From Avoided-Reinterventions Matter: A Budget-Impact Analysis Of The Eluvia Drug-Eluting Stent Across Different Time Horizons

**DOI:** 10.1017/S0266462325102201

**Published:** 2025-12-29

**Authors:** Thathya V Ariyaratne, Nishath Altaf, Bibombe Patrice Mwipatayi, Abimbola Williams, Virginia Priest

## Abstract

**Introduction:**

The need for reintervention peaks one to three years after an index endovascular intervention. Updating the Australian budget impact model becomes imperative to understand the impact of Eluvia™ drug-eluting stents (DES) over Zilver® PTX® drug-coated stents. This study forecasted the economic impact of Eluvia stents across various time horizons.

**Methods:**

Model inputs for clinical endpoints were obtained from the IMPERIAL trial results from three to five years, published sources, and publicly available data. A public healthcare payer perspective was adopted. Cost inputs were obtained from national cost averages in the National Hospital Cost Data Collection Public Sector Report 2020–21, to reflect post-COVID-19 cost of care. Population statistics were obtained from the Australian Bureau of Statistics to reflect the evolving demographics in Australia. The original model assumptions were unchanged, except for the annual procedure growth rate (3.2%).

**Results:**

Assuming an 80 percent endovascular procedural eligibility rate and a DES use rate of 10 to 28 percent (superficial femoral artery lesions), cumulative savings from avoided reinterventions for Eluvia DES were as follows: one-year, USD0.41 to 1.14 million; two-year, USD1.06 to 2.95 million; three-year, USD1.12 to 3.12 million; four-year, USD1.35 to 3.76 million; and five-year, USD1.58 to 4.39 million. When considering non-significant secondary trial endpoints, the total net savings were: one-year USD0.20 to 0.55 million; two-year USD0.37 to 1.03 million; three-year, USD0.68 to 1.88 million; four-year, USD1.16 to 3.22 million; and five-year, USD0.90 to 2.48 million. The cost savings from avoided reinterventions for Eluvia DES group, as a ratio of total net healthcare cost savings, was the highest during two-year horizon.

**Conclusions:**

Using Eluvia DES for treating peripheral artery disease offers substantial early savings to healthcare payers through avoided reinterventions. As such, a focus on clinical data during the reintervention risk peak at one to three years; one- to two-year budget cycles; and the use of high-quality devices upfront may improve patient outcomes and healthcare efficiencies.

